# Supplementary material for: Detailed molecular and epigenetic characterization of the pig IPEC-J2 and chicken SL-29 cell lines
Source: iScience. 2023 Feb 20;26(3):106252. doi: 10.1016/j.isci.2023.106252 (PMC10018572; doi:10.1016/j.isci.2023.106252)
Supplement: Data S1. Complete homer output for identified motifs in Pig IPECJ-2, related to Table 2 — Homer motif analysis results for histone modifications H3K4me1, H3K4me3, H3K27ac, and enhancer elements of pig IPECJ2 cell line. P-values >1e-10 are possible false positives. Within each folder (e.g. peak_files_CTCF) are the html files showing the identified motifs when using homer (e.g. homerResults.html). [file mmc2.zip › S5/Pig_IPECJ_2/peak_files_H3K4me3/homerResults/motif2.similar.html]

motif2

## Information for motif2

T
C
G
A
T
C
G
A
G
C
T
A
T
G
A
C
A
G
T
C
A
C
T
G
T
G
C
A
C
T
G
A
C
T
G
A
A
T
G
C
  
Reverse Opposite:  

A
T
C
G
A
G
C
T
A
G
C
T
A
C
G
T
A
G
T
C
C
T
A
G
A
C
T
G
C
G
A
T
A
G
C
T
A
G
C
T
  

|  |  |
| --- | --- |
| p-value: | 1e-287 |
| log p-value: | -6.616e+02 |
| Information Content per bp: | 1.699 |
| Number of Target Sequences with motif | 983.0 |
| Percentage of Target Sequences with motif | 4.45% |
| Number of Background Sequences with motif | 287.1 |
| Percentage of Background Sequences with motif | 1.09% |
| Average Position of motif in Targets | 149.0 +/- 85.1bp |
| Average Position of motif in Background | 139.9 +/- 101.2bp |
| Strand Bias (log2 ratio + to - strand density) | 0.1 |
| Multiplicity (# of sites on avg that occur together) | 1.06 |
| Motif File: | file (matrix) reverse opposite |

### Similar de novo motifs found

|  |  |  |  |  |  |  |  |
| --- | --- | --- | --- | --- | --- | --- | --- |
| Rank | Match Score | Redundant Motif | P-value | log P-value | % of Targets | % of Background | Motif file |
| 1 | 0.898 | T C A G C T G A G T C A C T G A T C A G G C A T T C A G T C G A C G T A C G T A T A C G G A C T C T A G | 1e-252 | -581.076949 | 2.70% | 0.45% | motif file (matrix) |
| 2 | 0.808 | A C G T G C A T G A C T A G T C T A C G A T C G A C G T C A G T | 1e-239 | -550.487932 | 29.78% | 20.38% | motif file (matrix) |
| 3 | 0.675 | T C A G G T A C A T C G G C T A T C G A G T C A T A G C G C A T | 1e-149 | -343.714667 | 29.35% | 21.84% | motif file (matrix) |
| 4 | 0.634 | A T C G G C A T A G C T A G C T T A G C G C T A A T G C G T A C A C T G A G C T | 1e-100 | -230.644202 | 27.07% | 21.04% | motif file (matrix) |
| 5 | 0.823 | A G T C C G T A C T G A C G T A T C G A A T G C T C A G C T G A C G T A C G T A A T G C A G T C T C G A | 1e-94 | -217.675146 | 0.29% | 0.01% | motif file (matrix) |
| 6 | 0.646 | C T A G T A C G A C G T A C G T G C A T A G T C G C T A A G T C A C G T A C G T A G T C G A C T A T C G | 1e-21 | -49.543463 | 0.25% | 0.05% | motif file (matrix) |
